# Professional Freedom

**Published:** 1907-10-26

**Authors:** 


### Editor's Letter-Box.

### "PROFESSIONAL FREEDOM."

We have received from Mr. Geo. W. F. Robbins, Secretary to the National Anti-Vivisection Hospital, a long letter in criticism of an article which appeared in our issue of October 5, under the title "Professional Freedom." Considerations of space forbid the publication of the letter *in extenso*, and there is the less need for this seeing that Mr. Robbins admits that "except for the apparently erroneous idea of our system at the Battersea General Hospital there is not much fault perhaps to be found with the article." The main object of the letter is presumably to negative the suggestion, alluded to in our article, that the medical officers of the Anti-Vivisection Hospital "are forbidden to make use of some kinds of treatment." But Mr. Robbins in his letter neither admits nor denies this proposition. He protests that "the freedom of judgment and action of our medical and surgical staff is . . . given full play," and that the declaration "we ask of an applicant is that he, of his own freely-formed and conscientious opinion is opposed to any experiments on living animals; . . . that he or she will not perform any such experiment, inoculation or operation known as vivisection; and will not aid or abet any other in so doing, and will also do all in his or her power to oppose the practice." Whether it is in the best interests of patients that the selection of medical officers to a hospital shall be restricted by the application of this test is a question upon which we do not here propose to enter. It is not the point at present in dispute. Upon the authority of the Council of the Hospital Sunday Fund it has been affirmed that by the rules of the Governing Body of the Anti-Vivisection Hospital the medical officers "are forbidden to make use of some kinds of treatment." Neither in his letter to us nor in the correspondence between himself and the Hospital Sunday Fund has Mr. Robbins contested the accuracy of this statement. This is the only point in our article upon which Mr. Robbins may reasonably claim to be heard, and so far it stands without challenge.—Editor, The Hospital.

### THE LONDON PROVIDENT DISPENSARIES COUNCIL.

*To the Editor of* The Hospital.

